# Assessing impact of exogenous features on biotic phenomena in the presence of strong spatial dependence: A lake sturgeon case study in natural stream settings

**DOI:** 10.1371/journal.pone.0204150

**Published:** 2018-12-05

**Authors:** Andrew O. Finley, Patrick S. Forsythe, James A. Crossman, Edward A. Baker, Kim T. Scribner

**Affiliations:** 1 Department of Forestry, Michigan State University, East Lansing, Michigan, United States of America; 2 Department of Natural and Applied Sciences, University of Wisconsin, Green Bay, Wisconsin, United States of America; 3 BC Hydro, Water License Requirements, Castlegar, British Columbia, Canada; 4 Michigan Department of Natural Resources, Fisheries Division, Marquette, Michigan, United States of America; 5 Department of Zoology, Michigan State University, East Lansing, Michigan, United States of America; 6 Department of Fisheries and Wildlife, Michigan State University, East Lansing, Michigan, United States of America; SOUTHWEST UNIVERSITY, CHINA

## Abstract

Modeling spatially explicit data provides a powerful approach to identify the effects of exogenous features associated with biological processes, including recruitment of stream fishes. However, the complex spatial and temporal dynamics of the stream and the species’ reproductive and early life stage behaviors present challenges to drawing valid inference using traditional regression models. In these settings it is often difficult to ensure the spatial independence among model residuals—a key assumption that must be met to ensure valid inference. We present statistical models capable of capturing complex residual anisotropic patterns through the addition of spatial random effects within an inferential framework that acknowledges uncertainty in the data and parameters. Proposed models are used to explore the impact of environmental variables on Lake sturgeon (*Acipenser fulvescens*) reproduction, particularly questions about patterns in egg deposition. Our results demonstrate the need to apply valid statistical methods to identify relationships between response variables, e.g., egg counts, across locations, and environmental covariates in the presence of strong and anisotropic autocorrelation in stream systems. The models may be applied to other settings where gamete distribution or, more generally, other biotic phenomena may be associated with spatially dynamic and anisotropic processes.

## Introduction

Ecology, hydrology, statistics, and interrelated subdisciplines in aquatic sciences are increasingly integrated to study complex relationships between stream physical features and the life histories of stream fishes at multiple spatial scales [1–3] that have important implications for management and conservation [2]. Research must be conducted in a spatially explicit fashion because stream hydrogeomorphological features are highly heterogeneous. Data are currently available for many fluvial systems (e.g., [4]) allowing studies of associations between aquatic physical data and biological phenomena (e.g., [3, 5]). However, practitioners face challenges developing and applying models capable of adequately accommodating non-Gaussian response variables and spatially complex autocorrelation, e.g., directional dependence, of physical and biotic variables within and among different watersheds and stream segments [1, 3, 6].

Spatial heterogeneity in physical stream characteristics associated with spawning means that eggs, during and after spawning event, will experience a range of environmental conditions that affect deposition and ultimately survival to subsequent development stages. From a management perspective, egg count is commonly used as a measure of year class strength; hence, there is a need to understand the relationships between the complex spatial and temporal dynamics of stream characteristics and reproductive output. It is, however, often challenging to develop valid statistical models to draw inferences about processes of interest from such complex systems—let alone assessing the extent to which findings can be extended to novel domains. For example, many standard parametric tests or model frameworks require data that is independent across space [7]. Failing to account for spatial correlation may result in inaccurate parameter estimates and erroneous conclusions regarding associations between environmental covariates and the response variable(s) of interest. Geostatisical methods attempt to account for spatially correlated residuals by including model components that estimate the characteristics of the underlying spatial process (see, e.g., [8, 9]).

Most available geostatisical models assume the relationship between point-referenced observations can be described using a function of Euclidean distance. Specific examples of such methods within the fisheries literature appear in classical kriging [10] and model-based survey design [11]. [12] extend these ideas to specify model random effects that accommodate both space and time dependence structures for modeling fish population dynamics.

Here, we define spatial regression models to quantify the effects of stream environmental covariates and the spatial dimensions of these effects associated with egg deposition within and among stream segments. We worked within a geostatistical setting, given that egg counts (the response) are point-referenced. Because spawning is confined to small stream segments, modeling spatial dependence using Euclidean distance is sensible. If observations were recorded along longer stream segments, or entire networks, then we would need to consider modeling approaches that are not strictly based on Euclidean distance to describe spatial dependence, see, e.g., [13–16]. Although we do not deal with network distance metrics, care is needed to specify models capable of accommodating complex residual patterns, e.g., anisotropy, through the addition of spatial random effects, and the use of inferential frameworks that acknowledge uncertainty in the data and parameters. The models proposed in this paper were developed to explore relationships between environmental variables and the deposition of gametes within and among spawning sites.

Many aspects of stream fishes reproductive ecology and physiology are tied to environmental variables associated with stream habitats [17]. Fishes often rely on exogenous cues to decide on the location and timing of reproduction [18–21]. Many fish species exhibit behaviors whereby parental decisions result in gametes being placed into a specific location (e.g., nest) characterized by a selected suite of environmental features [22, 23]. Other species, such as sturgeon (family *Acipenserformes*) are broadcast-spawners. For example, lake sturgeon (*Acipenser fulvescens*) are characterized by a promiscuous and aggregate mating system (Bruch and Binkowski 2002). Females are highly fecund (∼11,000 eggs/kg; [24]) and have adhesive eggs (2.7-3.8 mm diameter). Negatively buoyant eggs and sperm are released by groups of females and males into the water column. Eggs are distributed over broad areas and exposed to spatially heterogeneous environmental variables without post-ovulatory parental care. Reliance on environmental cues suggests that environmental variables that are associated with time and location of reproduction may be consistent across multiple spatial and temporal scales.

The lake sturgeon mating system and broad distribution of gametes creates a natural experimental setting to develop and illustrate models to investigate the effects of environmental variables on egg distribution. Because eggs from a single female are distributed passively over large expanses of stream habitat, micro-spatial heterogeneity in physical stream characteristics associated with the location of egg deposition means that eggs will experience a range of environmental conditions. Spawning time and location within and across years are characterized by differences in several environmental variables including temperature and river flow [20], which can be high during incubation [25]. High rates of egg mortality in other sturgeon species have been attributed to developmental arrest, predation, and physical stream processes that dislodge eggs [26, 27]. Features of stream habitat shown to be associated with egg loss include substrate [28, 29], ground and surface water exchange [30–32], and water velocity [29, 33].

The goals of this study were to provide insight into the: *i*) relationship between stream covariates and lake sturgeon egg deposition; *ii*) extent to which these relationships can be generalized to other locales and systems; and *iii*) level of model sophistication needed to draw valid inference about these relationships. Regarding the third point, we specifically wanted to know if failure to account for spatial structure, beyond that described by covariates, results in inaccurate characterization about the effect of covariates on egg deposition. To explore these questions, we developed spatial regression models that characterize egg deposition within different stream sections and allow us to quantify the effects of covariates that might affect the location of egg deposition.

## Materials and methods

### Study location

**Fig 1:**
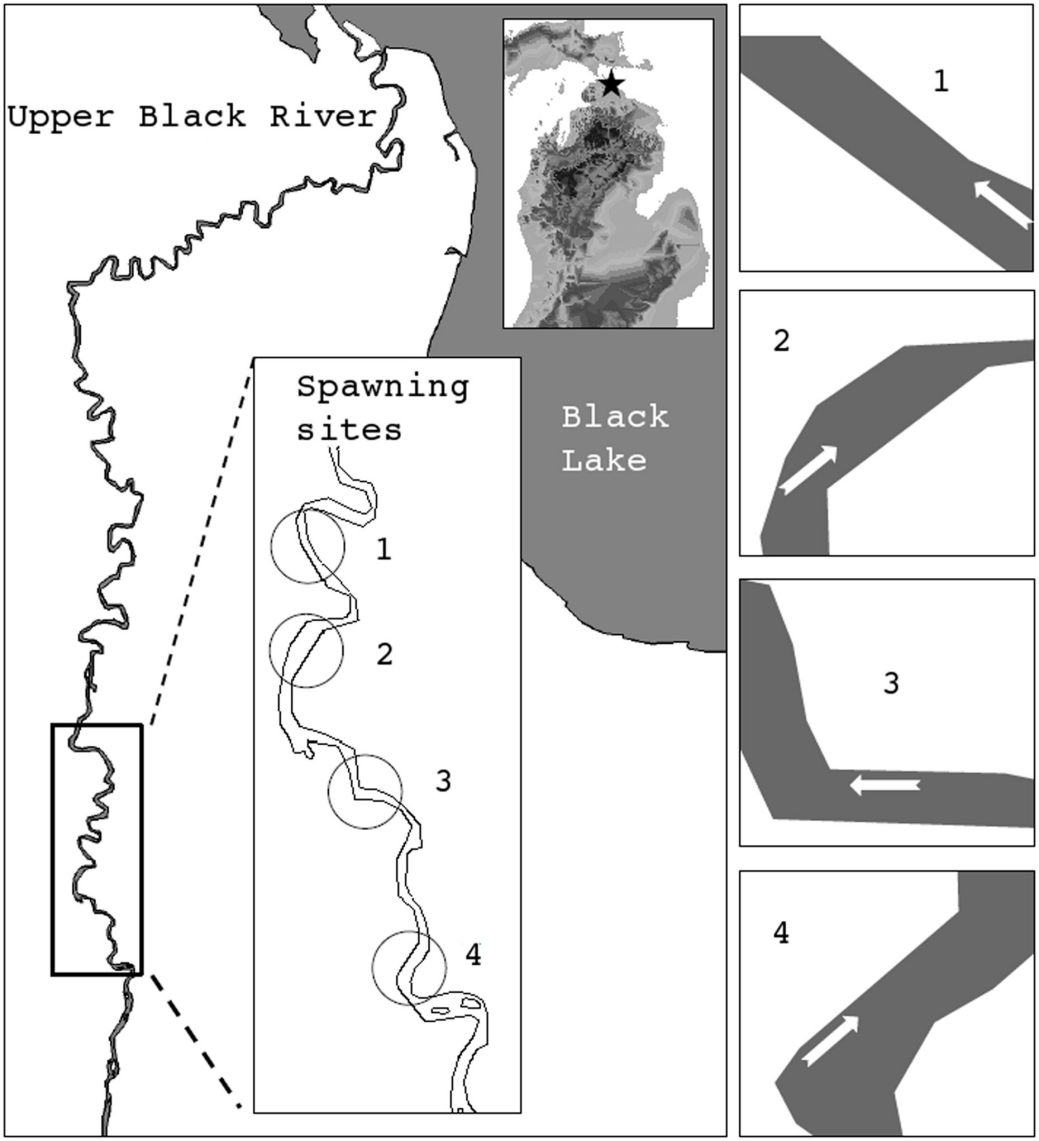
Left panel: star symbol on the inset map of Michigan indicates the Upper Black River study location. The inset box of the Upper Black River identifies the four spawning site locations with circle symbols. Right panels: provide a characterization of each spawning site with flow direction indicated with an arrow. River segment lengths over which measurements were taken are approximately 45, 25, 60, and 30 m, for Sites 1-4.

The models developed were applied to data from a well-studied lake sturgeon population that spawn in the Upper Black River (UBR), the primary tributary of Black Lake, MI (Fig 1). The UBR is relatively narrow and shallow with low average discharge. Spawning occurs within a shallow (<1.5 m) and wadable section (1.5 km) of the river. Within this section, individuals spawn during the spring (depending on the year between late April and early June) within six primary areas over a period of 28-45 days [21]. Seasonal patterns in water temperature and discharge are well characterized and have been shown to predict the timing and location of spawning activities [20, 21]. The stream hydrology at spawning sites allow unrestricted access to spawning adults, resulting in detailed observations of spawning behavior (e.g., spawning location, number of spawners, sex ratios, and spawning duration). Further, accessibility in the study site allows for the majority of the spawning population to be observed and captured at the spawning grounds. Enumeration of adult spawning aggregations also identifies the location of early life stage rearing habitats. Deposited eggs can be easily collected from stream substrates using sampling gear that is well suited to these stream conditions though not widely uses in larger rivers. Access to the stream also allows measurement of environmental covariates that might contribute to egg placement.

### Field sampling

**Fig 2:**
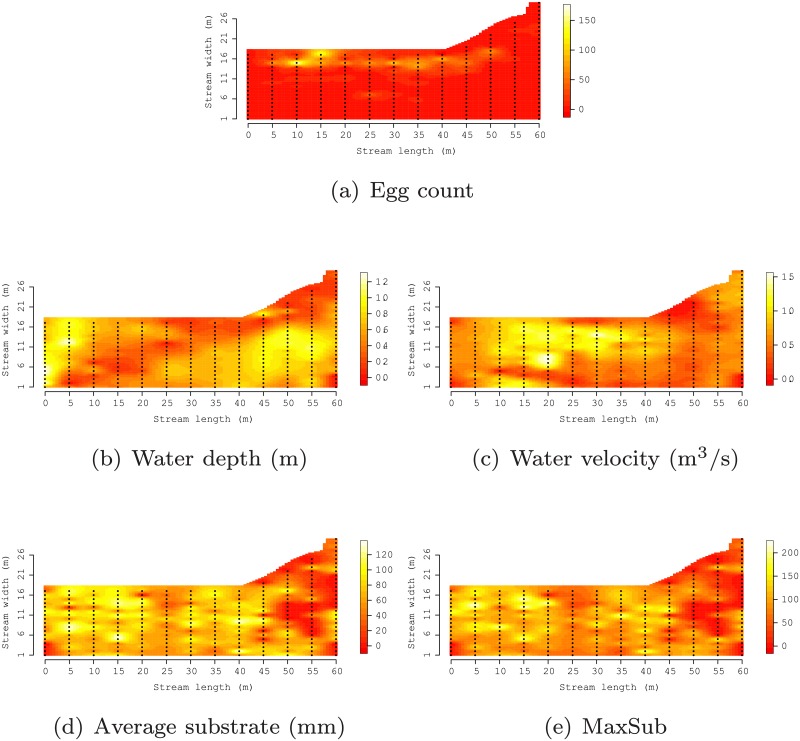
Point symbols indicate the locations where egg count and environmental covariates were measured at site 3. Underlying surfaces were generated by passing the given point values through an interpolator.

Surveys for spawning adults were conducted daily, starting upstream and moving downstream through the 1.5 km section of river. Upon capture, adults were tagged uniquely by sex with external floy tags (details in [27]). The location of each spawning aggregation was recorded using a handheld GPS and permanent stakes were placed along the stream bank. Data were collected at four spawning sites selected by spawning lake sturgeon in the UBR. Site 1 was sampled on 5/19/03, Sites 2 and 3 were sampled on 5/10/05 and 5/12/05, and Site 4 was sampled on 4/25/06, respectively (Fig 1). Transects were conducted across the stream channel starting ∼1 m below the observed spawning group and were repeated every 5 m downstream to cover the area of egg deposition. To ensure spawning had ceased and no new eggs were deposited, transects were conducted one day after spawning was completed and adults had left the site. Counts of eggs and measurements of environmental covariates were collected at a 1 m interval along each transect (see, e.g., Fig 2) using kick nets. Kick nets are a widely used approach to sample stream benthos including sturgeon [34] and have been used successfully to collect eggs in other studies (e.g., [35, 36]). Substrate was thoroughly disturbed for 5 seconds in the area (∼0.125 m^2^) immediately upstream of a small triangular net (305×305×305 mm opening). The kick net was held immediately down stream of the agitated area to ensure displaced materials were swept into the net. We assume the egg counts are proportional to the actual eggs present across the range of habitats encountered (deep vs shallow, coarse substrate vs fine). Materials collected by the kick net were emptied into white bottom trays and all contents were sorted and the number and status (live versus dead) of collected eggs were recorded.

Data on environmental covariates (water depth, water velocity, and substrate size) were collected concurrent with surveys of egg deposition. Stream depth (*Depth*) was recorded in meters using a stadia rod. Water velocity (*Velocity*) at the stream bed was recorded in cubic meters per second using an electromagnetic flow meter (Marsh-McBirney, Inc.). Average substrate size (*AvgSub*; mm length along the longest axis) was estimated using four stones collected from either the kick net sample or from the corresponding point on the transect. Maximum substrate size (*MaxSub*; mm) was estimated by measuring the largest stone collected within the transect point. Data available from the Dryad Digital Repository: https://doi.org/10.5061/dryad.tg706dv.

### Statistical analyses

#### Egg deposition

A Poisson regression model was used to relate egg count at sampling points along the transects and coinciding measures of environmental covariates. This model is adequate if spatial correlation in the response is completely explained by the covariates. Because lake sturgeon are broadcast spawners and eggs are extruded into a spatially complex fluvial environment, egg counts are expected to exhibit strong spatial dependence along the down-stream and across-stream sampling axes (i.e., similar egg counts in the proximate locations). Unmeasured covariates or covariates measured at the *wrong* scale can result in spatial dependence among the residuals, which violates the model assumptions. Therefore, to accommodate any lingering spatial structure, we include a spatially structured random effect in the model intensity. Given observations arise over a set of locations, say $S=\{s_{1},\ldots ,s_{n}\}$, where **s**_*i*_ is a sample location’s spatial coordinates, we specify the log-link model as:

$$\begin{array}{c} y\left(s_{i}\right)∼Poisson\left(\mu \left(s_{i}\right)\right);\;\log\left(\mu \left(s_{i}\right)\right)=x{\left(s_{i}\right)}^{\prime }\beta +w\left(s_{i}\right), \end{array}$$ (1)

where *y*(**s**_*i*_) is the *i*-th sample location egg count, which is assumed to be distributed (“∼”) as a Poisson variable with location specific conditional expectation *μ*(**s**_*i*_). This conditional expectation comprises vectors of covariates and associated slope parameters, **x**(**s**_*i*_) and ***β*** respectively, and *w*(**s**_*i*_) which provides local adjustment (with structured dependence) and captures the effect of unmeasured or unobserved spatial covariates.

We assume the random effects follow a zero mean Gaussian Process (GP), *w*(**s**) ∼ *GP*(0, *σ*^2^ *ρ*(·; ***θ***)), with variance *σ*^2^, and correlation function *ρ*(**s**, s*; ***θ***), where **s** and s* are two generic locations. The vector of parameters ***θ*** control the correlation function’s behavior. For a collection of *n* locations the *n* × 1 vector of random effects follows a Multivariate Normal distribution, **w** ∼ *MVN*(**0**, *σ*^2^*R*(***θ***)), where $R\left(\theta \right)={\left[\rho \left(s_{i},s_{j};\theta \right)\right]}_{i,j=1}^{n}$ is the correlation matrix. Spatial correlation functions of varying complexity are available for defining *ρ*(**s**, s*; ***θ***), see, e.g., [37]. A common choice for the correlation function is an isotropic exponential, *ρ*(**s**, s*; ***θ***) = exp(−*ϕ*∥**s** − s*∥), where *ϕ* is the spatial range parameter. Here it is assumed the spatial dependence is the same in all directions. Given the dynamics of the stream’s flow, isotropy is a rather unrealistic assumption. To explore the possibility of a more complex spatial structure we use an anisotropic form *ρ*(**s**, s*; ***θ***) = exp[−(**s** − s*)′ [*G*(*ψ*)Λ^2^*G*′(*ψ*)]^−1^(**s** − s*)]. Here, the rotation matrix *G*(*ψ*) controls directional dependence given the angle parameter *ψ* and the diagonal matrix Λ defines the rate of spatial decay along perpendicular axes. Specifically, the positive diagonal elements λ_0_ and λ_1_ corresponding to spatial decay parameters for the major and minor axes, respectively [38, 39], which in our situation corresponds to down stream vs cross stream, respectively.

When describing a spatial process it is useful to report the distance at which spatial dependence is negligible. This distance is referred to as the effective spatial range, and is defined here as the distance at which the spatial correlation drops to 0.05. For the isotropic exponential correlation function this is −log(0.05)/*ϕ* and for the anisotropic form this is −log(0.05)λ, where λ is a diagonal value in Λ. Note, there are two effective spatial ranges for each anisotropic process, one oriented with *ψ*, and the other perpendicular to *ψ*.

#### Implementation and model selection

The proposed models were fit following a Bayesian paradigm [40, 41]. As such, a prior distribution must be assigned to each parameter to complete the model specification. For all models the ***β***’s received *flat* prior distributions and the spatial variance parameters were assigned inverse-Gamma (*IG*) priors with hyperparameters *IG*(2, ⋅). This is considered a non-informative prior; with a shape value of 2, the *IG* distribution has infinite variance and is centered on the scale value, which is data set specific. The spatial range parameters λ’s and *ϕ*’s followed Uniform priors, which are chosen to support a spatial range from 0 to the maximum inter-location distance in the data set. The rotation parameters *ψ*’s also received a data set specific Uniform prior providing rotation estimates consistent with possible stream flow direction.

To compare several alternative models, we used the Deviance Information Criterion (DIC) [42]. Letting *Ω* be the generic set of parameters being estimated for each model (including random effects), we computed the expected posterior deviance $\bar{D(\Omega )}=E_{\Omega |Y}\left[-2\log L\left(Data|\;\Omega \right)\right]$, where *L*(*Data*|*Ω*) is the first stage likelihood from the respective model and the effective number of parameters (as a penalty) as $p_{D}=\bar{D(\Omega )}-D\left(\bar{\Omega }\right)$, where $\bar{\Omega }$ is the posterior mean of the model parameters. The DIC is then given by $\bar{D(\Omega )}+p_{D}$ and is easily computed from the posterior samples with lower values indicating better models.

For all models, three MCMC chains, with unique starting values, were run for 50,000 iterations. The CODA package in R (www.r-project.org) was used to diagnose convergence by monitoring mixing using Gelman-Rubin diagnostics and autocorrelations (see, e.g., [40]). For all analyses, acceptable convergence was diagnosed within 10,000 iterations (which were discarded as burn-in). The sampler was coded in C++ and Fortran and leveraged Intel’s Math Kernel Library threaded BLAS and LAPACK routines for matrix computations.

## Results

### Summary of spawning groups and environmental covariates

**Fig 3:**
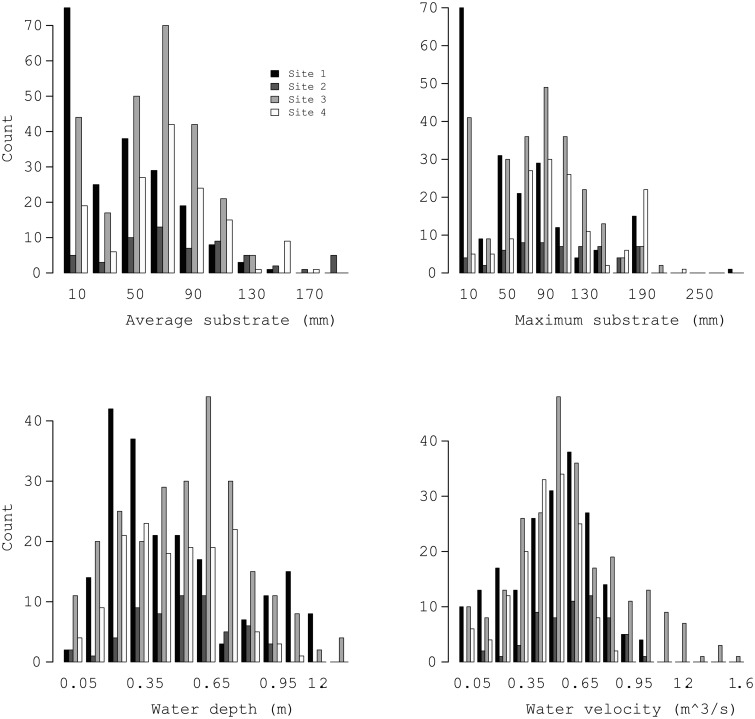
Frequency histograms for average substrate size, maximum substrate size, water depth, and water velocity for sites 1-4.

As illustrated in Fig 3, the four sampling sites exhibited similar ranges in environmental covariate characteristics including substrate sizes (1-200 mm), maximum substrate size (1-300 mm), water depth (0.1-1.6 m), and water velocity (0.1-1.6 m^3^/s). However, the distribution of values for each environmental covariate indicate that the physical features of areas where eggs were deposited varied considerably among sites, Fig 3. These distributions suggest Sites 1 and 3 have many sample locations with average and maximum substrate smaller than Sites 2 and 4. Also, Site 3 has several locations with water velocity greater than the other sites. Fig 2b–2e) offers a spatial representation of the distribution of these covariates for Site 3. Corresponding figures for Sites 1, 2, and 4, as well as figures for subsequent analysis results are provided as supplemental material.

### Embryo deposition and environmental covariates

A total of 9,426 eggs were collected across all spawning locations from 651 kick net samples. Egg counts varied across locations (Site 1 = 2,509, Site 2 = 564, Site 3 = 2,111, Site 4 = 4,242). Within each site, egg deposition exhibited non-random and spatially-dependent patterns, see, e.g., Fig 2(a), often occurring in discrete patches. Eggs were primarily deposited along the downstream axis on the side of the river where spawning took place and from 0 to 60 m downstream from spawning locations.

Both the non-spatial and spatial versions of Model (1) were fit using observations from the 198, 60, 249, and 144 sampling locations surveyed at Sites 1-4, respectively. Table 1 provides candidate models’ parameter estimates for each site. Looking first at the spatial model’s effective spatial range parameter estimates, λ_0_ and λ_1_, we see there is relatively strong but variable spatial patterns among the residuals. For example, Site 2 exhibits the shortest effective spatial range of ∼5 m, whereas Site 3 shows the longest range of ∼54 m. For Sites 1, 3, and 4, the disparity between λ_0_ and λ_1_ suggests there are strong anisotropic patterns in spatial dependence and hence an isotropic spatial covariance function would not be appropriate. In each of these sites *ψ* describes the orientation of the major spatial range parameter axis λ_0_. Here we present *ψ* in both radians and degrees, where in both cases zero is perpendicular to the west river bank. Not surprisingly, parameter estimates of λ_1_ suggest a shorter range of spatial dependence perpendicular to water flow. Sites with the maximum egg dispersal distance, e.g., Site 3, also had the highest average water velocity.

**Table 1:** Summary of egg deposition non-spatial and spatial models’ parameter estimates for each site. Parameter posterior credible intervals, 50 (2.5 97.5) percentiles. Those *β* parameters that are significant at the 0.05 level are bolded.

	Site 1		Site 2	
Parameters	Non-spatial	Spatial	Non-spatial	Spatial
***β***_0_	**1.86 (1.71, 1.99)**	-1.94 (-4.61, 0.43)	**1.42 (0.90, 1.93)**	-0.27 (-2.54, 2.04)
***β***_*Depth*_	**-0.44 (-0.61, -0.28)**	**1.89 (0.11, 3.84)**	**0.96 (0.33, 1.39)**	0.87 (-2.18, 3.44)
***β***_*Velocity*_	-0.04 (-0.19, 0.15)	1.18 (-0.21, 2.60)	**-0.79 (-1.29, -0.35)**	-0.20 (-2.84, 2.25)
***β***_*AvgSub*_	**0.014 (0.012, 0.015)**	0.004 (-0.005, 0.013)	**-0.012 (-0.014, -0.009)**	-0.015 (-0.034, 0.001)
***β***_*MaxSub*_	**0.003 (0.002, 0.004)**	**0.007 (0.002, 0.012)**	**0.015 (0.012, 0.018)**	**0.022 (0.008, 0.039)**
*σ*^2^	–	5.19 (2.96, 9.56)	–	2.42 (1.36, 4.93)
*ψ* degree	–	10.78 (-36.31, 39.46)	–	-14.49 (-43.19, 43.08)
λ_0_	–	10.96 (5.00, 16.10)	–	2.23 (0.37, 9.51)
λ_1_	–	5.66 (2.94, 11.65)	–	1.83 (0.39, 9.29)
Eff. range (−log(0.05)λ_0_)	–	32.85 (14.98, 48.25)	–	6.68 (1.13, 28.50)
Eff. range (−log(0.05)λ_1_)	–	16.97 (8.81, 34.91)	–	5.48 (1.17, 27.85)
DIC	-8710	-14115	-1548	-2139
*p*_*D*_	5	117.4	5.7	48.5
	Site 3		Site 4	
Parameters	Non-spatial	Spatial	Non-spatial	Spatial
***β***_0_	0.02 (-0.12, 0.25)	**-3.49 (-5.79, -0.41)**	**0.87 (0.72, 1.01)**	**-4.20 (-7.02, -0.81)**
***β***_*Depth*_	**-0.34 (-0.58, -0.18)**	0.68 (-1.36, 2.82)	**0.81 (0.62, 0.93)**	**7.26 (2.05, 9.16)**
***β***_*Velocity*_	**1.71 (1.57, 1.87)**	0.22 (-1.02, 1.50)	**1.78 (1.58, 1.96)**	**2.64 (0.42, 4.42)**
***β***_*AvgSub*_	**0.016 (0.013, 0.019)**	**0.019 (0.005, 0.035)**	**0.009 (0.008, 0.010)**	0.002 (-0.004, 0.009)
***β***_*MaxSub*_	-0.001 (-0.003, 0.001)	-0.009 (-0.020, 0.002)	**0.003 (0.003, 0.005)**	-0.002 (-0.007, 0.003)
*σ*^2^	–	10.43 (6.33, 17.35)	–	5.84 (3.54, 10.51)
*ψ* degree	–	5.88 (-7.44, 19.98)	–	16.94 (-8.51, 64.34)
λ_0_	–	18.12 (10.84, 22.05)	–	10.33 (5.87, 12.14)
λ_1_	–	6.30 (3.71, 10.40)	–	5.045 (2.83, 10.31)
Eff. range (−log(0.05)λ_0_)	–	54.29 (32.48, 66.07)	–	30.97 (17.60, 36.37)
Eff. range (−log(0.05)λ_1_)	–	18.89 (11.13, 31.18)	–	15.11 (8.47, 30.89)
DIC	-6163	-11080	-22051	-28620
*p*_*D*_	5	117.1	5.1	95.4

The relatively long effective spatial ranges as well as the magnitude of the spatial variance parameter values, i.e., *σ*^2^, suggest that even after accounting for the covariates there is substantial unexplained spatial pattern in the number of eggs within the spawning sites. The presence of this residual spatial autocorrelation violates an assumption of the non-spatial equivalent to Model (1). As noted in Section 1, this violation can result in erroneous estimates of the regression coefficients associated with the covariates.

**Fig 4:**
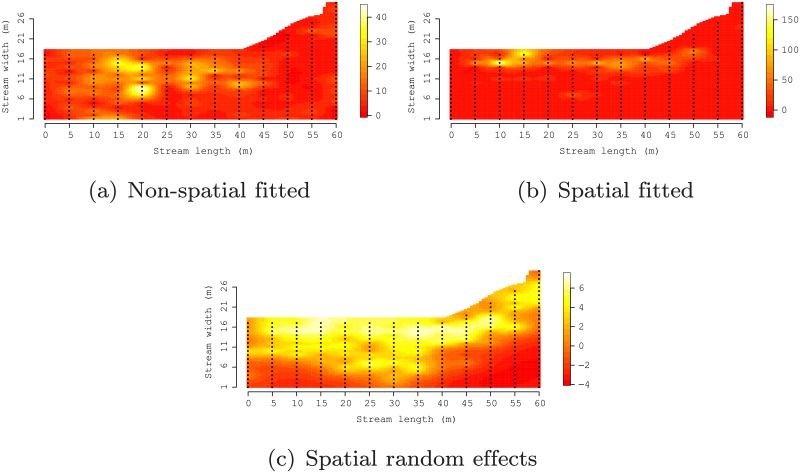
Interpolated surfaces of fitted values from the non-spatial and spatial models (a) and (b), respectfully, and spatial model random effects (c) for site 3. These fitted values can be compared to the observed egg counts illustrated in Fig 2(a).

Comparison between the non-spatial and spatial model regression coefficients, i.e., *β*’s, in Table 1 shows that ignoring the residual autocorrelation does in fact result in very different conclusions about the association between the covariates and egg deposition. For example, in Site 1 the non-spatial model estimates the median and 95% credible interval (CI) for *β*_*Depth*_ at -0.44 (-0.61, -0.28), which excludes zero and can hence be considered statistically significant at a 0.05 level. This estimate suggests that water depth is negatively associated with the number of eggs deposited. The relationship between depth and number of eggs changes to positive and significant after model assumptions are satisfied through the addition of the spatial random effects. This is presumably a more accurate portrayal of the system. The non-spatial model identifies more covariates as significant than the spatial model across all sites. Further, once the residual space dependence is accommodated, there is little consistency in regression coefficient across the sites. For instance, the spatial model parameter estimates suggest water depth is significant and positive for Sites 1 and 4, but not in Sites 2 and 3. Maximum substrate size is significant in Sites 1 and 2 suggesting that larger or coarser substrate is positively associated with number of eggs deposited—a relationship that does not hold for Sites 3 and 4. In addition to meeting model assumptions, and presumably providing a more accurate view of the relationship between deposition and the environmental covariates, the spatial random effects in Model (1) improves fit to the data as reflected in the consistently lower DIC values given in Table 1. The larger effective number of parameters, *p*_*D*_, for the spatial models versus the non-spatial model, is due to the addition of the random effects. The fit of the non-spatial and spatial models is illustrated in Fig 4(a) and 4(b), respectively. These surfaces can be compared to the observed values illustrated in Fig 2(a) and shows the spatial model does, in fact, more closely approximate the distribution of eggs across the stream segment. Fig 4(c) provides the spatial random effects surface that illustrates where local adjustment to *μ*(**s**) is needed to fit the observed data after accounting for the covariates.

## Discussion

Following from Section 1, the non-spatial models’ parameter estimates provided misleading conclusions about the relationship between deposition and the covariates. Of 16 environmental covariates measured across sites (i.e., 4 covariates times 4 sites), 14 were found to be significant predictors of egg deposition based on the non-spatial model, compared with only six when using the more statistically appropriate spatial models, Table 1. Several lines of evidence including measures of model fit (DIC, pD; Table 1) and the magnitude of spatial range parameters demonstrate that without accounting for spatial dependencies, predictions of egg deposition, and subsequent uses of these predictions, would be inaccurate and misleading from a biological or management perspective.

The spatial models showed that lake sturgeon egg depositional profiles were spatially autocorrelated and highly anisotropic across all spawning sites. Comparison of spatial range parameters revealed that spatial dependence was always greater in the downstream than across the stream direction. Environmental covariates did not show consistent significant impact across sites and generally did not explain a substantial portion of responses’ variability. Rather, the spatial random effects explained the majority of spatial variability in egg distribution. Importantly, results underscores the need to work within modeling frameworks capable of capturing residual spatial dependence if we wish to draw valid inference about those covariates that are included in the model. Examples of egg deposition studies that have failed to use spatial models are common (see, e.g., review by [43]). Failure to incorporate spatial models can mis-direct efforts to use direct estimates of total egg counts as a measure of recruitment potential [44]. Comparisons of the actual egg depositional surface (Fig 2(a)) to spatially and non-spatially fitted egg surfaces in Fig 4 clearly show that if egg count estimates are based on non-spatial models, results should be interpreted with caution. In dynamic stream environments, estimates based on a sample of available habitat should not be extrapolated to larger areas to estimate total reproductive effort (total numbers of eggs).

Despite the specificity of spawning habitats typically selected by lake sturgeon [45], the known importance of “high quality” habitats [17], the spatial model results suggested considerable heterogeneity across sites in the relative magnitude and direction of effects of stream environmental variates on egg depositional patterns. Assumptions that adult reliance on environmental cues to time and select locations for reproduction imply that effects of environmental covariates would be comparable across multiple spatial and temporal scales. Adult behavioral plasticity in spawning site selection (e.g., four sites sampled in this study) resulted in heterogeneous egg depositional profiles across sites dictated by site-specific environmental covariates.

Spatial dependence is likely due to the species’ spawning behavior and the physical system [45, 46]. Eggs are released and subjected to site-specific fluvial dynamics near the stream bed that non-randomly determine the direction of egg drift and the distance traveled. Model results suggest some environmental covariates are useful for explaining patterns in egg deposition, supporting our prediction that hydro-geomophological environmental stream variables will explain some variability in egg deposition within a site. Specifically, egg deposition was significantly associated with substrate maximum and average size. Water velocity and depth were also found to be significant predictors of egg deposition. Our results are concordant with other studies that found stream variables including water depth, water velocity, and substrate size are predictive of egg deposition for migratory fishes [47–49]. The magnitude and direction of association between numbers of lake sturgeon eggs deposited with substrate size, depth, and velocity reported in other studies (e.g., [50, 51]) might differ if spatially explicit models were considered. The effect of flow to generate “hot spots” has been widely described in other aquatic organisms (e.g., macroinvertebrates) and is frequently observed in river systems [52]. Our results are consistent with other studies of behaviors such as nest site selection in birds and in migratory fishes which exhibit clustered or patchy distributions due to abiotic (i.e., habitat) specificity related to the species reproductive ecology [53, 54]. Our proposed models, can be used in future studies to quantify the impact of additional spawning behavior and physical system covariates that might help explain patterns seen in the spatial random effects. Potentially useful covariates that influence where eggs ultimately settle include flow at the substrate subsurface, hyporheic discharge, and other microhabitat characteristics (e.g., interstitial spacing or substrate embeddedness).

## Summary

Properly linking components of the physical and biotic environment to currencies of reproductive performance is important to understand population, species, and community responses to anthropogenic change and abilities of natural systems to deliver ecosystem services [55–57]. Our study demonstrates the need to employ appropriate statistical methods to properly elucidate relationships between response and environmental covariates in the presence of strong and anisotropic autocorrelation in complex stream systems. As demonstrated here, ignoring spatial dependence can result in falsely precise estimates of regression coefficients associated with environmental covariates and erroneous predictions of egg deposition. If estimates are based on non-spatial models, results should be interpreted with caution. In dynamic stream environments, estimates based on a sample of available habitat should not be extrapolated to larger areas to estimate total reproductive effort (total numbers of eggs).

Most commonly applied regression models require the residuals, i.e., after accounting for covariates, to be independent and identically distributed. In many settings the biotic phenomena of interest exhibit spatial dependence that cannot be completely explained by covariates. Exploratory analyses of non-spatial model residuals using surface plots, e.g., Fig 4, or variograms (if the response is Gaussian) will typically reveal if there is lingering spatial structure and hence if a spatial random effect is needed. As shown in this study, residual spatial patterns can exhibit directional dependence, in which cases an anisotropic spatial correlation function should used. Failing to account for spatial correlation can result in inaccurate parameter estimates and erroneous conclusions regarding associations between environmental covariates and the response variable(s) of interest.

## Supporting information

S1 File Data and results for sites 1, 2, and 4. (PDF)
