# Supplementary material for: Assessing impact of exogenous features on biotic phenomena in the presence of strong spatial dependence: A lake sturgeon case study in natural stream settings
Source: PLoS One. 2018 Dec 5;13(12):e0204150. doi: 10.1371/journal.pone.0204150 (PMC6281228; doi:10.1371/journal.pone.0204150)
Supplement: S1 File — (PDF) [file pone.0204150.s001.pdf]

Supplementary figures for "Assessing impact of exogenous features on biotic phenomena in the presence of strong spatial dependence: A lake sturgeon case study in natural stream settings"

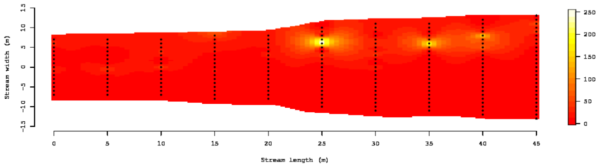

(a) Egg count

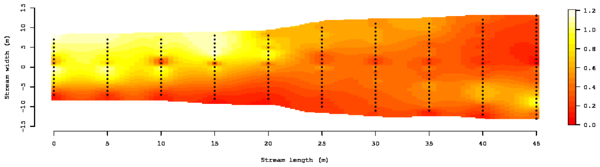

(b) Water depth (m)

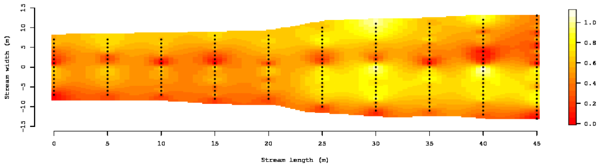

(c) Water velocity (m^3/s)

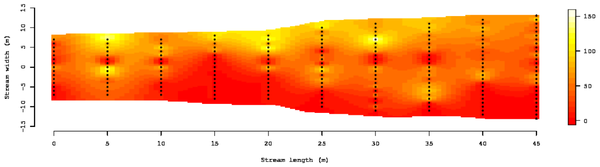

(d) Average substrate (mm)

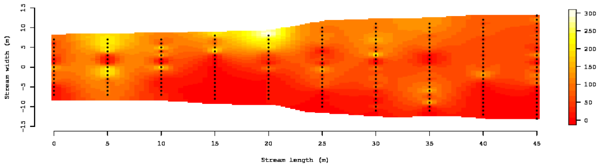

(e) MaxSub

S1 Fig: Point symbols indicate the locations where egg count and environmental covariates were measured at Site 1. Underlying surfaces were generated by passing the given point values through an interpolator.

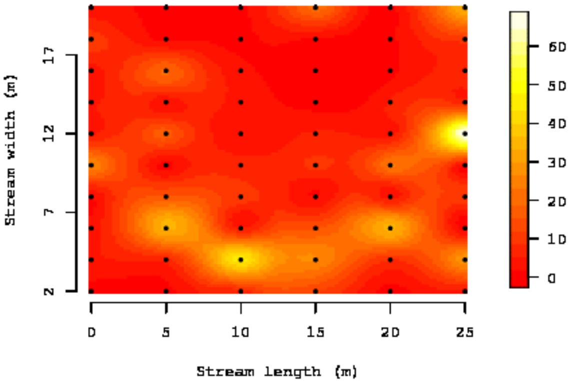

(a) Egg count

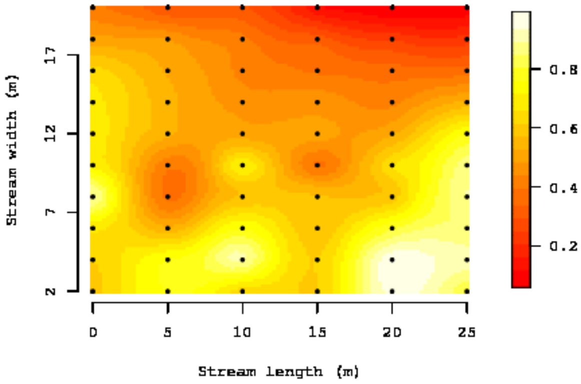

(b) Water depth (m)

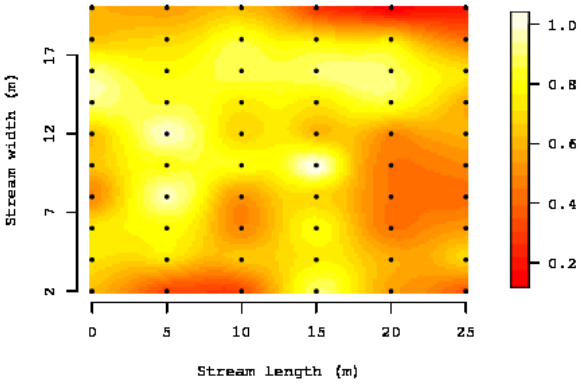

(c) Water velocity (m^3/s)

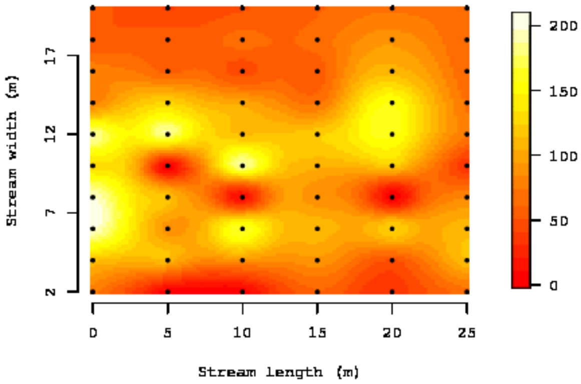

(d) Average substrate (mm)

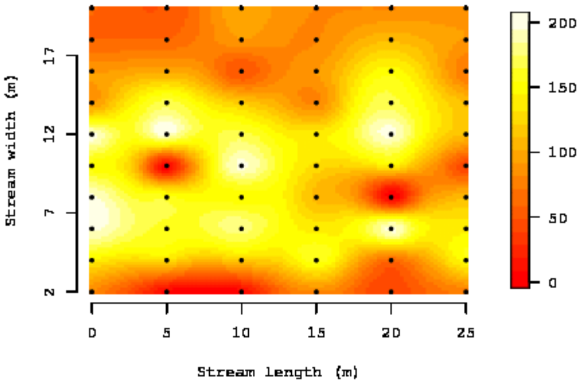

(e) MaxSub

S2 Fig: Point symbols indicate the locations where egg count and environmental covariates were measured at Site 2. Underlying surfaces were generated by passing the given point values through an interpolator.

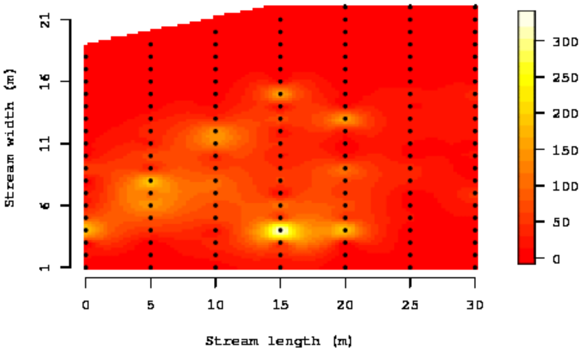

(a) Egg count

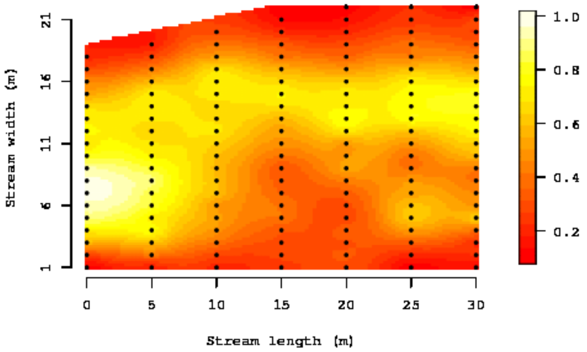

(b) Water depth (m)

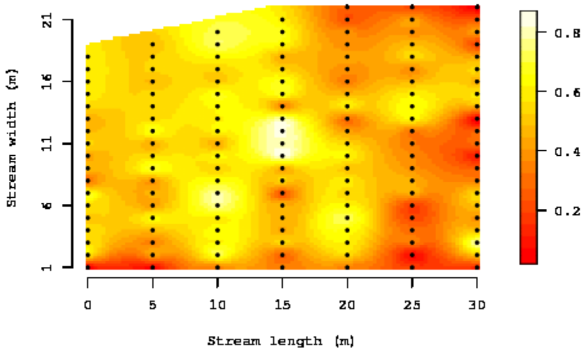

(c) Water velocity (m^3/s)

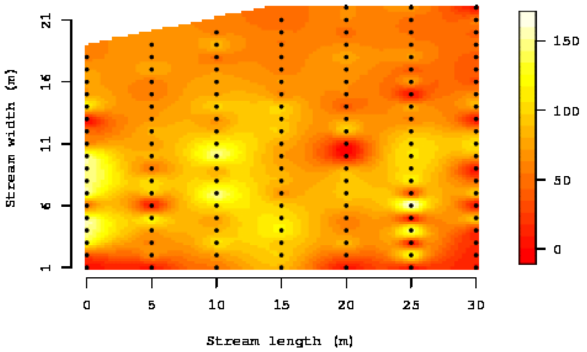

(d) Average substrate (mm)

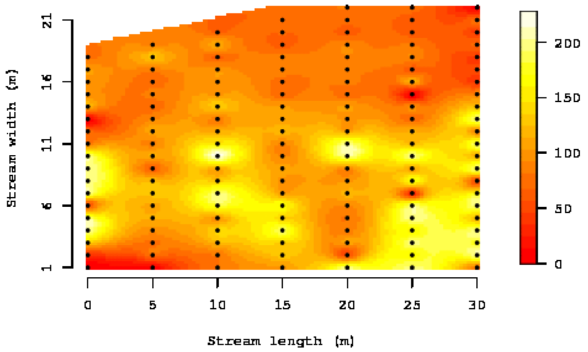

(e) MaxSub

S3 Fig: Point symbols indicate the locations where egg count and environmental covariates were measured at Site 4. Underlying surfaces were generated by passing the given point values through an interpolator.

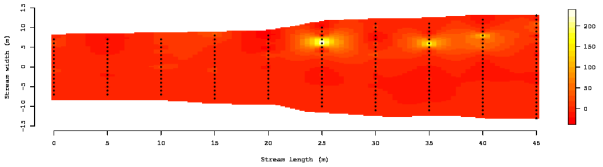

(a) Non-spatial residuals

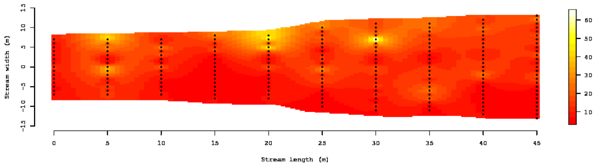

(b) Non-spatial fitted

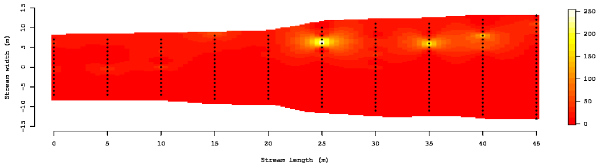

(c) Spatial fitted

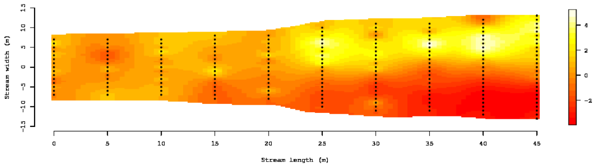

(d) Spatial random effects

S4 Fig: Interpolated surfaces of residual and fitted values from the non-spatial model (a) and (b), respectfully, and spatial model fitted and random effects (c) and (d), respectively, for Site 1.

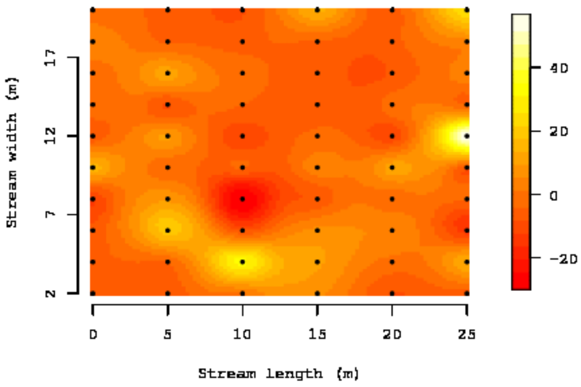

(a) Non-spatial residuals

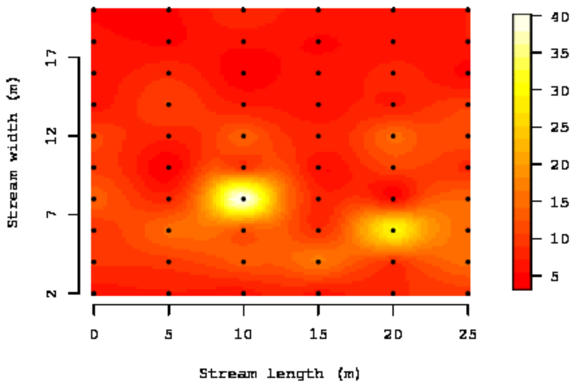

(b) Non-spatial fitted

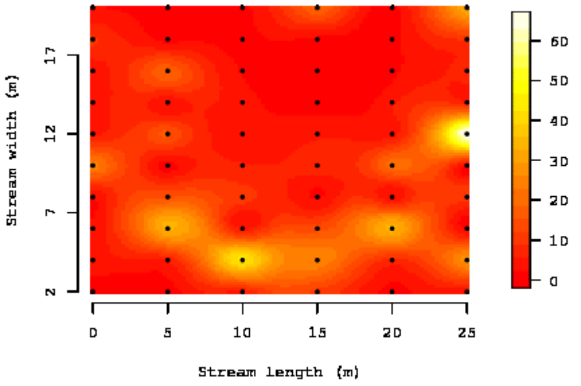

(c) Spatial fitted

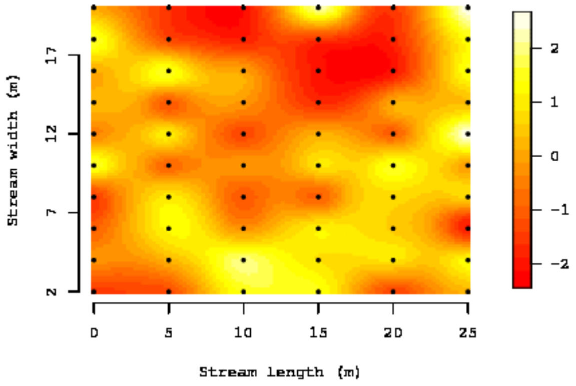

(d) Spatial random effects

S5 Fig: Interpolated surfaces of residual and fitted values from the non-spatial model (a) and (b), respectfully, and spatial model fitted and random effects (c) and (d), respectively, for Site 2.

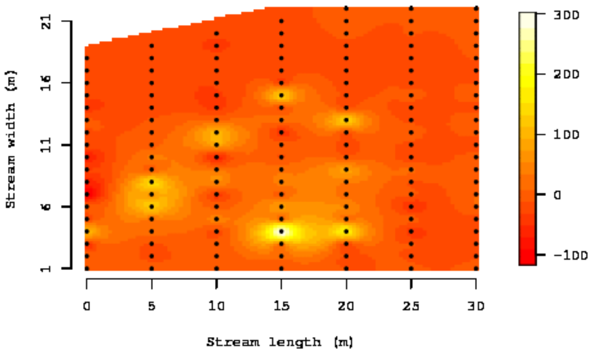

(a) Non-spatial residuals

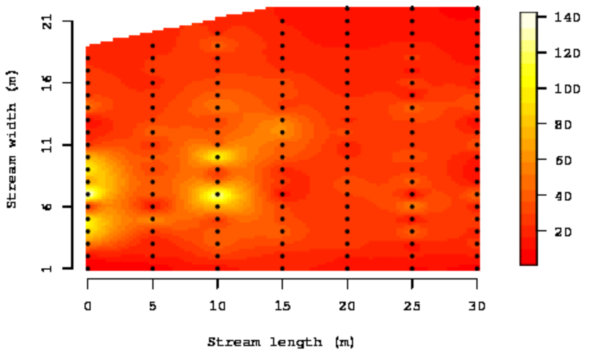

(b) Non-spatial fitted

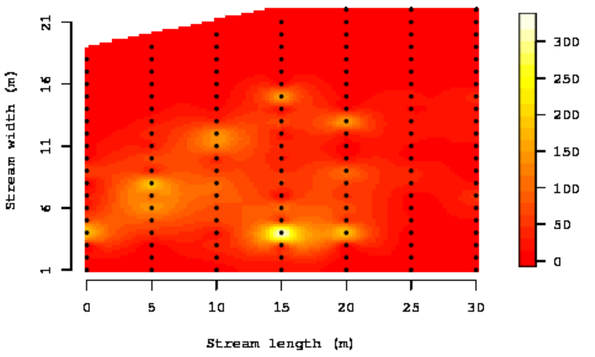

(c) Spatial fitted

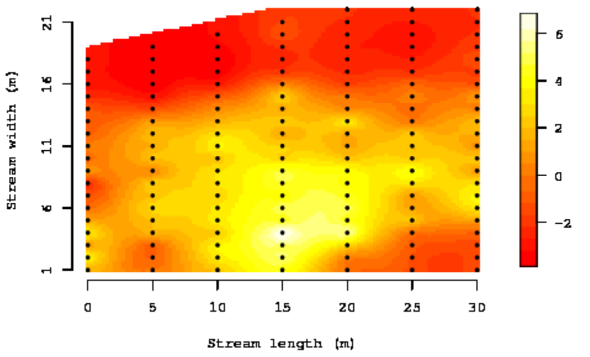

(d) Spatial random effects

S6 Fig: Interpolated surfaces of residual and fitted values from the non-spatial model (a) and (b), respectfully, and spatial model fitted and random effects (c) and (d), respectively, for Site 4.
